# Breathing-Focused Yoga Intervention on Respiratory Decline in Chronically Pesticide-Exposed Farmers: A Randomized Controlled Trial

**DOI:** 10.3389/fmed.2022.807612

**Published:** 2022-03-11

**Authors:** Vipin Dhansoia, Vijaya Majumdar, N. K. Manjunath, Usha Singh Gaharwar, Deepeshwar Singh

**Affiliations:** ^1^Swami Vivekananda Yoga Anusandhana Samsthana, Bengaluru, India; ^2^School of Environmental Sciences, Jawaharlal Nehru University, New Delhi, India; ^3^Swami Shraddhanand College, University of Delhi, Alipur, Delhi

**Keywords:** farmers, pesticide exposure, breathing-focused yoga intervention, respiratory decline, cognitive decline

## Abstract

**Background:**

Occupational exposure to pesticides has been associated with lung and cognitive function exacerbations. In the present study, we tested the effectiveness of breathing focused yoga intervention on alleviation of adverse respiratory and cognitive effects associated with chronic pesticide exposure in farmers.

**Methods:**

We undertook a parallel, two-armed randomized controlled trial with blinded outcome assessors on a chronically pesticide-exposed farming population. The study was conducted at district Panipat, State Haryana located in the Northern part of India from November 2019 to August 2020. A total of 634 farmers were screened, and 140 farmers were randomized to breathing-focused yoga intervention (BFY, *n* = 70) and waitlist control arms (*n* = 65). BFY was delivered weekly in 45-min group sessions over 12 weeks followed by home-based practice. The primary outcome was the change in spirometry-based markers of pulmonary function from baseline expressed as raw values, Global Lung Initiative (GLI) percent predicted (pp), and GLI z-scores after 24 weeks of intervention. Secondary variables were Trail making tests (TMT A and B), Digit symbol substitution (DSST), and WHO Quality of life-BREF (WHOQOL-Bref). Analysis was by intention-to-treat. Mediation analysis was done considering oxidative stress markers as potential mediators.

**Results:**

At the end of 6 months of intervention, the overall follow-up in the participants was 87.85% (*n* = 123); 90% (*n* = 63) in the control group, and 85.71% in the yoga group (*n* = 60). The mean age of the study cohort (*n* = 140) was 38.75 (SD = 7.50) years. Compared with the control group, at 24 weeks post-intervention, the BFY group had significantly improved status of the raw sand z scores markers of airway obstruction, after adjusting for confounders, FEV1, FVC, FEF25-75 [z score-adjusted mean differences (95% CI); 1.66 (1.10–2.21) 1.88 (1.21–2.55), and 6.85 (5.12–8.57), respectively. A fraction of FEF25-75 change (mediation percentage 23.95%) was explained by glutathione augmentation. There were also significant improvements in cognitive scores of DSST, TMT-A and TMT-B, and WHOQOL-Bref.

**Conclusion:**

In conclusion, regular practice of BFY could improve the exacerbations in the markers of airway obstruction in chronically pesticide-exposed farmers and cognitive variables. A significant mediating effect of glutathione augmentation was also observed concerning the effect of the intervention on FEF25-75. These findings provide an important piece of beneficial evidence of the breathing-based yoga intervention that needs validation across different farming ethnicities.

**Clinical Trial Registration:**www.ClinicalTrials.gov, identifier: CTRI/2019/11/021989.

## Introduction

Pesticide use is an integral measure for agricultural sustainability, one of the primary objectives of the sustainable development goals (SDG-2) (1). However, the large-scale use of pesticides has surfaced as a double-edged sword associated with a varying range of detrimental health outcomes (2–15). Prevention of work-related respiratory disease constitutes the primary focus of the National Institute of Occupational Safety & Health (NIOSH) (16). Though the modifiability of occupational exposures through educational strategies has grabbed some clinical interest as a preventive measure for further exacerbations including chronic obstructive pulmonary disease (COPD), and chronic bronchitis (17). However, these interventions require changing the behavior of farmers which has been notified as a difficult outcome to achieve given the observation that many protective recommendations are never adopted by farmers (17).

Adverse respiratory consequences expressed as reductions in spirometric variables [forced expiratory volume in 1 s (FEV1), forced vital capacity (FVC), and their ratio percentage FEV/FVC%] are the most widely reported health concerns of chronic pesticide exposure (3–9). These manifestations are the established risk factors for fixed airway obstruction including chronic obstructive pulmonary disease (6). Several lines of evidence support the beneficial effects of yoga-based interventions on the respiratory system in various non-clinical and clinical settings exacerbations such as COPD and asthma (18–25). The improved efficiency of respiratory function associated with yoga practice has been attributed to various factors including enhanced ventilatory functions, increased forced vital capacity, FEV1, maximum breathing capacity and breath-holding time, maximal stretching of respiratory muscles, efficient use of diaphragmatic and abdominal muscle, blunting of excitatory pathways regulating respiratory systems, etc. (20, 22–25). Explicitly there is a particular indication of the limited effectiveness of the yoga-based intervention to its breathing-focused practices as compared to yoga postures against critical manifestations such as COPD (19). These respiratory exercises are relatively simple, low cost, and could be incorporated into the daily lives of farmers. However, there is no clinical trial report available addressing the effectiveness of these practices in pesticide-exposed farmers with adverse respiratory manifestations. Further, given the notion that the efficacy of yoga-based interventions depends on the fitness levels of the individuals (21), the generalisability of findings from different subject populations is limited.

Cognitive impairment is another major health exacerbation of chronic pesticide exposure. It is a risk factor for neurodegenerative diseases (13, 14) and could underline the reduced well-being of farmers directly linked to the sustainability of agriculture (26) and hence, calling for clinical attention. Several studies support role of yoga as an effective intervention to enhance cognitive function (Hedges' *g* = 0.33, standard error = 0.08, 95% CI = 0.18–0.48), with the strongest effects reported for attention and processing speed (*g* = 0.29, *p* < 0.001), followed by executive function (*g* = 0.27, *p* = 0.001) and memory (g = 0.18, *p* = 0.051) (27, 28). Importantly, these domains of cognition also intersect with pesticide exposure-induced cognitive decline, we thereby hypothesized that farmers with pesticide exposure will benefit cognitively through yoga-based interventions.

In view of the lack of available studies focused on the management of adverse chronic health effects in pesticide exposed farmers, we conducted a randomized clinical trial to test if 24 weeks of regular breathing-focused yoga practice could alleviate their adverse respiratory and cognitive manifestations against a wait-list control group.

Over recent years, there has been increased recognition of the importance of evaluating hypothesized mediating mechanisms in clinical trials (29). Oxidative stress is one of the unanimous pathological mechanisms underlying pesticide-induced toxicity of various pesticides (30–32), with lipid peroxidation and GSH depletion being the critical modulators of airway damage in obstructive lung diseases (33). Alleviation of imbalances in oxidative stress parameters has been one of the mechanistic insights obtained from yoga-based clinical research (34–36). Hence, the present trial also aimed to test the mediating role of the oxidative stress markers underlying the effectiveness of the breathing-focused yoga intervention on the respiratory and cognitive outcomes.

## Methods

### Study Design

**Figure 1:**
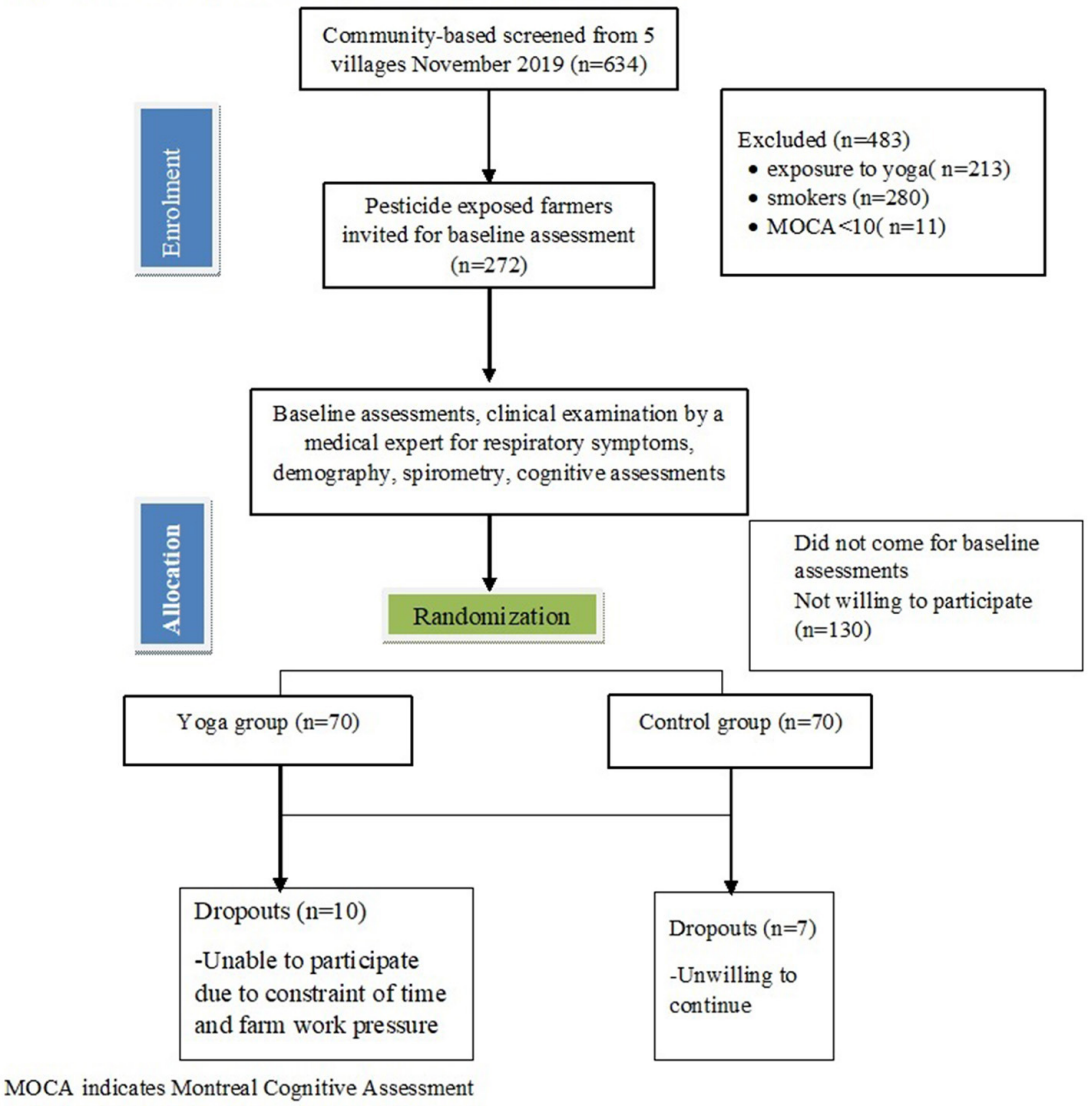
Trial consolidated standards of reporting trials profile. MOCA indicates Montreal Cognitive Assessment.

The study was a two-armed, randomized, parallel-group clinical trial with breathing-focused yoga intervention and the wait-list control groups with blinded outcome assessors (Figure 1). Details of the same have been appended in the study protocol (Supplementary Material). The trial was conducted at district Panipat, State Haryana located in the Northern part of India from November 2019 to August 2020. Farmers were invited to participate and were recruited during the meetings conducted by the village organizations. Only one member from each household was randomly selected to avoid any within-family clustering effects. After a detailed explanation of the study objectives and design, informed consent was obtained from willing individuals. The study was conducted following the CONSORT statement for non-pharmacological interventions and was approved by the Institutional ethics committee. The study was also registered with clinical trials of India registration number: CTRI/2019/11/021989.

### Participants

The participants were male farmers of the age group between 18 and 49 years, naïve to the practice of pranayama or other yoga-based practices, and with at least 6 months of self-reported spraying operations in the field. Farmers with prior exposure to yoga or any other mind-body medicine, symptoms of acute pesticide exposure/poisoning, smokers/ex-smokers, self-reported diagnosis of respiratory disease (such as COPD, asthma, bronchiectasis, pulmonary fibrosis, etc.), history of chronic or terminal disorders (such as active cancer, severe heart or cerebrovascular disease), or any limitations that could have led to difficulties in follow-up or assessments (such as mental illness or severe cognitive impairment, Montreal cognitive assessment, MoCA score <10) (37). were excluded from the study. For additional details see Supplementary Table 1.

### Randomization and Blinding

An external statistician, not directly involved in the implementation of the BFY had randomized the participants during their baseline visit in a 1:1 ratio (*n* = 70, each arm) using a sequence randomizer. The allocation sequences were sealed and participants were informed about the further process immediately after their baseline assessment. Owing to the nature of the intervention, blinding was not possible, however, outcome measures were blinded for the randomization groups.

### Intervention

All the participants of the yoga group followed a breathing-focused yoga module for 24 weeks. For the initial 12 weeks, the instructions for the yoga practices were given by certified yoga teachers for 45 min for 6 days/week. Following the same, participants were advised to do daily home-based practice for the next 12 weeks; this was done to integrate the intervention into their daily routine settings. The farmers were not restricted from doing their routine farm work on fields and thereby were obligatorily physically active. The intervention included physical practices (loosening practices, breathing practices with body movements, asanas), relaxation techniques, pranayama, lectures regarding the importance of yoga, lifestyle changes through notional corrections, the importance of wearing personal protective equipment during pesticide spray. Since farmers were involved in physically-demanding routine activities and based on the indicative relevance of breathing-focused yoga interventions on pulmonary function under various settings, the intervention was drafted with special emphasis on breathing practices, relaxation techniques, and meditation (20, 28). Asansa (physical postures) (pavanamuktasana, sukhasana, gomukhasana, paschimotanasana, and vakrasana) were included only for preparatory requiremrnts for the practice of pranayama. Further, under pranayama, Bhastrika pranayama was included based on the associated beneficial outcomes on lung function as well as on cognitive improvement (20, 28). The pranayama session was drafted as a comprehensive respiratory exercise regime of 25 min, composed of fast practices Kapalabhati interspersed with Surya bedhana (20). Details of intervention are presented in Supplementary Table 2 of the Study Protocol.

### Waitlist Control Group

For inactive control participants, we chose a wait-list design as we deemed it as an ethically appropriate alternative to provide needed care to the control pesticide-exposed group following the trial. Though the subjects in the wait-list group participated in no active intervention, while recruitment, they were instructed to continue their daily activities (without engaging in regular structured exercise) and were also given weekly once group lectures focused on the importance of wearing personal protective equipment during pesticide spray. All subjects received monthly phone calls to assess for any subjective changes in health. After the completion of the 24th week study, these participants received the same yoga-based intervention given to the intervention group post their data collection.

### Outcomes

All outcome assessments were done at baseline and 6 months. Standard measures of spirometry included forced vital capacity (FVC), forced expiratory volume in one second (FEV1), the ratio of forced expiratory volume in 1 s to forced vital capacity (FEV1/FVC), forced expiratory flow between 25 and 75% of the FVC, FEF25–75 and peak expiratory flow rate (PEFR). The primary outcome was the adjusted mean difference in lung function variables analyzed as spirometric data from baseline to the 24^th^ week. The data was presented as raw spirometric scores. Additionally, in order to meet the worldwide diagnostic standard, free of bias due to age, height, sex and ethnic groups, we used the Global Lung Function prediction equations to derive percent predicted values and standard deviation (z-) scores adjusted for sex, age, and height and ethnicity (38, 39). As specific reference ranges do not yet exist for South Asian population, the Caucasian equations (i.e., derived from white subjects of European origin) were used to derive the Global Lung Function Initiative (GLI) based scores. The secondary outcome variables were changes in cognitive functions scored through Digit Symbol Substitution Test (DSST) and Trail Making Tests part A and B (TMT-A and B); and psychological variables scored through perceived stress scale (PSS), and World Health Organization Quality of Life–BREF (WHOQOL-Bref). The neurocognitive tests/domains were selected based on the previous reports on neuropsychological outcomes in pesticide exposed farmers (13, 14). Mitigation of oxidative stress was hypothesized as the causal mediation mechanism for the breathing focused yoga and hence, the planned mediation analysis included oxidative stress markers; Malondialdehyde (MDA), Superoxide dismutase (SOD), and Glutathione (GSH).

### Assessments

Baseline assessments of study outcome measures were performed before subjects were randomized. Assessments were repeated at the end of 6 months of intervention. The preliminary information was obtained from all study subjects which included questions on demographic data, and those related to pesticide exposure including detailed exposure information, names of the pesticides used, mode of application, period, dose, frequency of pesticide applications, and personal protective equipment repair status, duration used, etc.

### Respiratory Parameters

A pulmonary function test was performed to assess pulmonary impairment in pesticide sprayers by using a spirometer (RMS Helios-702, India) following the standards of lung function testing of the American thoracic society/European respiratory Society (ATS/ERS) (40). Standard measures of spirometry included forced vital capacity (FVC), forced expiratory volume in 1 s (FEV1), the ratio of forced expiratory volume in 1 s to forced vital capacity (FEV1/FVC), forced expiratory flow between 25 and 75% of the FVC (FEF25–75%) and peak expiratory flow rate (PEFR). Participants were instructed to breathe, and three reproducible measurements each of FEV1, FVC, and maximal mid-expiratory flow were obtained. The highest values were documented and used for analysis. Other spirometric variables, including forced expiratory flow at 25–75% (FEF25–75) and peak expiratory flow (PEF), were obtained from the trial with the highest combined FEV1 and FVC. Using the Excel macro for GLI, reference values, the lower limit of normal (LLN), Z-scores, and percentiles for FEV1, FVC, and the FEV1/FVC ratio were calculated for each subject in the reference population available from www.lungfunction.org (41, 42). Height and weight at the time of spirometry were measured to the nearest 0.1 cm on a stadiometer and 0.1 kg on an electronic scale, respectively.

### Neurocognitive Parameters

The Montreal Cognitive Assessment (MoCA) was used to evaluate the overall cognitive abilities of the participants (37). Cognitive function was assessed using the neuropsychological tests, DSST (43, 44) (for executive function, speed of processing, attention), and Trail Making Test A/B (TMT-A: speed of processing; TMT-B: executive function) (45–48). DSST is a component of the Wechsler Adult Intelligence Test with high test-retest reliability. This pen and pencil-based test has a considerable executive function component, evaluates psychomotor speed, attention, and executive function. The subject was given a key grid of numbers and matching symbols and a test section with numbers and empty boxes. The test consists of filling as many empty boxes as possible with a symbol matching each number. The score is the number of correct number-symbol matches achieved in 90 s. We used the DSST scores as a continuous variable. TMT measures scanning and visuomotor tracking, divided attention, and cognitive flexibility. Two raw scores (time needed to complete TMT A and TMT B) and three derived scores (TMT B-A, TMT B/A, and TMT (B-A)/A) were calculated for each participant. These tests were selected based on the previous reports on neuropsychological outcomes in pesticide-exposed farmers.

### Psychological Assessments

Stress perception was assessed using the perceived stress scale (PSS), a 10-item well-validated scale that gauges chronic stress on a 40-point scale (49). A total score ranging from 0 to 40 is computed by reverse scoring the four positively worded items and then summing all the scale items. Higher scores indicate greater levels of perceived stress. Though not as diagnostic criteria, PSS scores of 0–13, 14–26, and 27–40 points have been considered as indicators of low, moderate, and high perceived stress, respectively (50).

The quality of life (QOL) of the participants was assessed using the World Health Organization Quality of Life – BREF (WHOQOL-Bref) (51), a standardized comprehensive instrument comprising 26 items that elicits the perceived physical health, psychological health, social relations and environment—related QOL in an individual.

### Biomarkers of Oxidative Stress

Oxidative stress markers, reduced glutathione (GSH) were estimated in the whole blood whereas, TBARS (Thiobarbituric acid reactive substances), and SOD (Superoxide dismutase) were analyzed from the hemolysate. TBARS concentration was expressed as serum malondialdehyde (MDA). The plasma and the buffy coat were removed from whole blood by centrifugation at 2,000 rpm for 10 min at 4°C. The red cells were washed thrice with normal saline and a hemolysate(s) was prepared as follows: MDA levels were measured with the method described by Ohkawa et al. (52). The plasma and the buffy coat were removed from whole blood by centrifugation at 2,000 rpm for 10 min at 4°C. The red cells were washed thrice with normal saline and a hemolysate(s) was prepared as follows: For the estimation, MDA hemolysate was prepared by mixing 1.9 ml of cold distilled water with 0.1 ml of packed cell volume (PCV) suspension. For estimation of SOD activity: The remaining red cells were haemolysed by approximately adding 1.5 volumes.

### Statistical Analysis

Given the lack of reported minimally clinically significant difference suggested for FEV1 defined for clinical trial endpoints for occupationally impaired lung function. The calculated sample size of *n* = 140 was based on the reported effect of on FEV1 [effect size of 0.54, (123/ml) improvement] 20 for 80% power and a 2-sided α = 0.05, with assumed attrition of 20% over 6 months. To meet the objective of recruiting 140 subjects, a rough sampling frame of 500 households was generated. The distribution of continuous variables was analyzed for normal distribution (using the Kolmogorov–Smirnov statistic) and for homogeneity of variance (Levene's test). Data for these variables are shown as means and standard deviation (SD). Covariates considered were age, educational level, BMI, cumulative exposure index (CEI), and serum achetylcholinesatse levels. Algorithms for calculation of CEI are Given in Supplementary Table 3. All statistical analyses were performed blinded to the randomization group and results are reported using intention-to-treat analysis. the 2012 Global Lung Function Initiative (GLI) reference equations were used and percent predicted and z-scores were calculated, using the open-source GLI R Macro. The GLI Z-score is a standardized measure of the positioning of an observed measurement in the distribution of the population from which the GLI reference values are derived and takes both between-subject and age- and height-related variability into account. LLN was defined as the lower fifth percentile in the distribution from which the GLI reference values are derived, as calculated by the GLI Excel macro. Airway obstruction was defined as FEV1/FVC less than the lower limit of normal as per the recommendations of The American Thoracic Society (ATS)/European Respiratory Society (ERS) (53). Linear regression was used to analyze study outcomes as adjusted mean differences (AMDs), additionally adjusted for their comparable value at baseline and other covariates Missing data were minimal. A *P*-value < 0.05 was considered to indicate significant differences between adjusted means.

We applied causal mediation method, to investigate if oxidative stress could be a causal pathway between intervention and the outcome. We fit mediation models to estimate the direct and indirect effects of the intervention assuming a mediating effect of the oxidative stress markers. Mediation analysis was performed using the methods described by Valeri and Vanderweele (54) to investigate direct and indirect effects of the BFY on study outcomes at 6 months. The PROCESS SPSS Macro version 2.13, model four was used to perform analysis by fitting a linear regression model to the outcomes with yoga yreatment and the mediators included were the covariates (described above), and then fitting a regression model to the mediator (linear or logistic depending on the mediator) including intervention as a covariate. In mediation analysis, effects can be broken down into separate paths: the c path between the treatment and outcome (without accounting for potential mediators), the a path between the intervention and the potential mediator; and the b path between the potential mediator and the outcome (Supplementary Figure 1). The mediating (indirect) pathway is calculated as the product of paths a and b (ab). Univariable linear regression models were fitted to the potential mediators MDA, GSH and SOD to test whether there was an association between the BFY and the mediators. Since a variable can only be a mediator of treatment if there is a significant effect (*p* < 0.05) of treatment on the mediator (path a), Following the sam, linear regression analyses were performed to examine the relationships between treatment allocation and change in each of the potential mediators, and between change in each of the potential mediators and the outcome posttreatment scores.

## Results

### Flow of Patients

The flow of patients into the study is shown in Figure 1. During the months of November-January 2019–20, we had screened 634 farmers from five nearby villages of Panipat district state Haryana, India. Out of 634 farmers screened, only 280 fitted the eligibility criteria (Figure 1), of which only 140 completed the baseline assessments who were randomized into yoga and control groups. A total of 130 participants (92.85%) completed the post-intervention assessment.

### Demographics at Baseline

The mean age of the study cohort (*n* = 140) was 38.75 (SD =7.50) years; and their mean BMI was 22.44 (SD = 1.37) kg/m^2^ (Table 1). Mean pesticide exposure among sprayers was found to be 5.71 (SD = 3.04) years. As compared with participants, non-participants were of lower age and had comparatively less exposure to pesticides (Supplementary Table 4). All the study subjects belonged to agricultural occupation with similar socioeconomic status (data not shown) with mean period of education as 3.54 (2.77) years. Aligining with the previous observations, farmers seemed to be exposed to combination of multiple pesticides, mostly organophosphates (see Supplementary Table 5) with mean serum cholinesterase levels of 5.37 (SD = 0.88) matching their exposure status (55). Table 1 also demonstrates the distribution of the spirometric variables following conversion to the GLI z-scores. Notably, the median z-score values were well-below zero [FEV1 = −3.39 (1.36); FVC = −3.07 (1.60); FEV1/FVC = −1.73 (1.76); FEF25–75 = −1.73 1.76, mean (SD)]. The median FEV1 z-score was less than −1.64, the lower limit of normal and the median FVC and FEF25–75 z-scores approached this mark. Almost the entire cohort had mild cognitive impairment (98.6%, MOCA scores 18–25). At baseline, the distribution of the demographic and study variables were found to be fairly even with the non-significant differences between the study groups (*p* > 0.05) (for details, see Table 1), except for DSST and TMT scores. However, the distribution of global cognition was balanced between the groups (MoCA, *P* = 0.225). The farmers were also exposed to a mixture of various pesticides, mostly organophosphate and a cumulative effect of pesticides, measured by activity of serum cholenesstase activity levels aligned with the range observed in previous populations with similar duration of pesticide exposure. Though almost the entire cohort exhibited potentially unsafe behavior with respect to the use personal protective equipments use with 72.85% reported none. There was also a significantly skewed distribution of PPE use between the study groups (Supplementary Table 6), however, the cumulative pesticide exposure index was equally distributed between the groups.

**Table 1:** Baseline characteristics of study participants.

**Variables**	**Overall (*n* = 140)**	**Yoga (*n* = 70)**	**Control (*n* = 70)**	***P*-value**
Age (yr)	38.75 (7.50)	37.64 (8.31)	39.86 (6.47)	0.081
Height (m)	1.73 (0.05)	1.74 (0.05)	1.73 (0.05)	0.481
Weight (Kg)	67.55 (5.07)	68.36 (5.51)	66.74 (4.47)	0.060
BMI (Kg/m^2^)	22.44 (1.37)	22.63 (1.45)	22.25 (1.27)	0.100
Education, (years)	3.54 (2.77)	3.93 (3.35)	3.16 (1.99)	0.100
Pesticide exposure in years (yr)	5.71 (3.04)	6.28(3.93)	5.15 (3.04)	0.061
Serum cholinesterase (KU/ml)	5.37 (0.88)	5.30 (0.94)	5.44 (0.82)	0.355
Cumulative pesticide exposure index (CEI)	8125.13 (6022.36)	8670.10 (5782.09)	7587.9 (6244.98)	0.291
***Adverse respiratory symptoms, n (%)***				
Wheezing	22 (15.7)	14 (20.0)	8 (11.4)	0.164
Dry cough	12(8.6)	9 (12.9)	3 (4.3)	0.128
Productive cough	106 (75.7)	54 (77.1)	52 (74.3)	0.693
Dyspnoea	98 (70)	57 (81.4)	41(58.6)	0.001*
Airflow obstruction	79 (56.29)	44 (62.85)	35 (49.23)	0.078
***Lung function characteristics***				
FEV1 (L), mean (SD)	2.06 (0.70)	2.04 (0.76)	2.08 (0.64)	0.691
FEV1 GLI PP	54.91 (18.80)	55.43 (17.11)	54.34 (20.58)	0.738
FVC (L), mean (SD)	2.92 (0.82)	2.79 (0.87)	3.04 (0.74)	0.065
FVC GLI PP	64.27 (18.28)	66.47 (16.73)	61.90 (19.68)	0.147
FEV1/FVC GLI PP	85.46 (15.21)	83.39 (15.23)	87.69 (15.00)	0.167
PEFR Pred (%)	43.76 (19.02)	44.03 (21.17)	43.49 (16.77)	0.867
FEF25-75 Pred (%)	49.71 (23.82)	45.44 (19.62)	53.99 (26.85)	0.03
***Z-Scores***				
FEV1 (L) z-score	−3.39 (1.36)	−3.36 (1.21)	−3.41 (1.50)	0.846
FVC (L) z-score	−3.07 (1.60)	−2.88 (1.46)	−3.27 (1.73)	0.158
FEV1/FVC z-score	−1.73 (1.76)	−1.98 (1.66)	−1.46 (1.84)	0.090
FEF25–75% z-score	17.23 (5.20)	16.83 (5.25)	17.66 (5.17)	0.355
***Cognitive function***				
MoCA score	22.31(1.95)	22.11(2.10)	22.51 (1.77)	0.225
DSST score (s)	38.34 (8.38)	35.63 (7.60)	41.04 (8.30)	<0.001*
TMT-A (s)	43.92 (18.81.216)	34.53 (16.44)	53.31(16.34)	<0.001*
TMT-B (s)	104.72 (42.61)	92.56 (37.20)	116.89 (44.43)	<0.001*
***Secondary variables***				
PSS	22.57 (5.95)	22.80 (5.89)	22.34 (6.04)	0.651
WHOQOL-Bref	47.61 (11.11)	43.97 (11.02)	51.24 (10.03)	<0.001*
***Oxidative stress indices***				
MDA, nmol/l	2.57 (7.66)	2.481(7.36)	2.66 (7.70)	0.153
GSH, mg/ml	2.51 (0.73)	2.61 (0.62)	2.46 (0.80)	0.083
SOD (units/min/mg protein)	0.23 (0.13)	0.24 (0.13)	0.22 (0.13)	0.410

*Continuous variables are represented as means (SD), and categorical variables are represented as number (%); s stands for seconds, t = independent samples t-test statistic; and χ^2^ = Chi-Square test statistic. Cumulative pesticide exposure index CEI; Airflow obstruction was defined as FEV1/FVC less than the lower limit of normal as per the recommendations of The American Thoracic Society (ATS)/European Respiratory Society (ERS). defined as FVC, forced vital capacity; FEV1: forced expiratory volume in 1 s; FEF25-75: forced expiratory flow; GLI PP, percent predicted values of FEV1, FVC and FEV1/FVC derived using Global Lung Function Initiative (GLI) equations; z-scores are standard deviation scores of spirometric variables adjusted for sex, age, and height using the Global Lung Function Initiative (GLI)-2012 equations; MOCA, Montreal Cognitive Assessment; DSST, Digit symbol substitution test; TMT-A, Trail making test A; TMT-B, Trail making test B; PSS, Perceived stress score; World Health Organization Quality of Life – BREF (WHOQOL-Bref); MDA, Malondialdehyde; GSH, Glutathione; Superoxide dismutase, SOD*.

### Primary Outcomes

At the end of 6 months of intervention, the overall follow-up in the participants was 87.85% (*n* = 123); 90% (*n* = 63) in the control group, and 85.71% in the yoga group (*n* = 60). The adjusted means of the all spirometric variables and their z-scores are presented in Table 2. In the intention-to-treat analysis with the raw spirometric data on the 140 randomized patients, BFY group had a significantly improvement in FEV1 (L) [AMD, 1.02, 95% CI (0.75–1.38), *p* < 0.001)], FVC (L) [AMD, 1.14 95% CI (0.79–1.49), *p* < 0.001], FEF25–75 [AMD, 29.33 95% CI (22.46–36.20) *p* < 0.001], PEFR [AMD, 43.47 95% CI (35.33–51.60), *p* < 0.001] as compared to the controls, following adjustment for age, height, education level, cumulative pesticide exposure, and serum cholineestase levels. However, no significant between group difference was observed for FEV1/FVC% (Table 2). Analyses of z-scores which are independent of age, and height, gave similar results (FEV1 AMD = 1.66 (95% CI = 1.10–2.21), FVC AMD = 1.88 (95% CI = 1.21–2.55) FEV1/FVC GLI pp AMD = 3.19 (95%CI=−8.68–14.96), and FEF25–75 z-score AMD=6.85 (95% CI = 5.12–8.57) following adjustment for education level, cumulative pesticide exposure and serum cholinesterase levels.

**Table 2:** Outcome measures (primary and secondary) after 6 months of follow-up.

**Outcomes**	**Adjusted means (Yoga) mean (SE)**	**Adjusted means (control) mean (SE)**	**AMD (95% CI)**	***F* value, partial eta square**
**Primary**				
***Lung function***				
FEV1 (L)	3.02 (0.09)	1.99 (0.09)	1.02 (0.75–1.29)^**^	56.84, 0.33^**^
GLIFEV1 PP	68.80 (3.73)	48.19 (3.73)	20.61 (10.05–31.165)^**^	14.92, 0.11^**^
FVC (L)	3.81 (0.12)	2.65 (0.12)	1.14 (0.79–1.49)^**^	41.81, 0.26^**^
GLIFVC PP	72.04 (3.99)	52.31 (3.99)	−19.75 (8.40–31.10)^*^	11.87, 0.087^*^
GLIFEV1/FVCPP	81.40 (4.15)	84.54 (4.15)	3.19 (−8.68–14.96), 0.600	299.14, 0.002
FEF25-75 (L.sec-1)	76.66 (2.43)	47.34 (2.37)	29.33 (22.46–36.20)^**^	71.46, 0.38^**^
PEFR	93.88(2.88)	49.81 (2.80)	43.47 (35.33–51.60)^**^	111.91,0.49^**^
FEV1 (L) z-score	−1.38 (0.20)	−3.03 (0.20)	1.66 (1.10–2.21)^**^	34.61, 0.218^**^
FVC (L) z-score	−1.25 (0.23)	−3.13 (0.23)	1.88 (1.21–2.55)^**^	31.04, 0.200^**^
FEV1/FVC z-score	−0.59 (0.15)	−0.59 (0.15)	0.01 (−0.44–0.45), 0.982P	0.00, 0.00
FEF25–75% z-score	25.75 (0.61)	18.90 (0.60)	6.85 (5.12–8.57)^**^	62.01, 0.37^**^
**Secondary**				
***Cognitive function***				
DSST	49.27 (1.01)	37.44 (0.98)	11.82 (8.90–14.75)^**^	64.15, 0.36^**^
TMT A (s)	24.18 (1.18)	49.00 (1.14)	−24.60 (−28.14–21.05)^**^	189.06, 0.62^**^
TMT B (s)	71.68 (2.68)	113.67 (2.61)	−41.99 (−49.72– −34.25)^**^	115.69, 0.50^**^
***Psychological well-being***				
PSS	18.77 (0.66)	20.25 (0.64)	−1.47 (−3.33–0.38)	2.45, 0.021, 0.12
WHOQOL-Bref	64.45(1.37)	37.20 (1.34)	27.25 (23.27–31.23)^**^	183.69, 0.62^**^
***Oxidative stress markers***				
MDA, nmol/ml nmol/ml	1.90 (1.00)	2.53 (0.98)	−63.72 (−91.94–35.05)	20.03, 0.15^**^
SOD (units/min/mg protein)	0.32 (0.02)	0.26 (0.18)	0.06 (0.010–0.11)	5.38, 0.04, 02^*^
GSH, mg/ml	3.55 (0.10)	2.48 (0.10)	1.08 (0.79–1.37)	53.48, 0.32^**^

*FVC, forced vital capacity; FEV1, forced expiratory volume in 1 s; GLIFEV1PP - indicates Global Lung Function Initiative (GLI) percent predicted of FEV1, FEF25–75, forced expiratory flow; FVC, forced vital capacity; FEV1 (L), forced expiratory volume in one second (L indicates liter); FEV1/FVC, ratio of forced expiratory volume in one second to forced vital capacity; FEF (25–75), forced expiratory flow between the 25 and 75% of the FVC; PEFR, peak expiratory flow rate; z-scores are standard deviation scores of spirometric variables adjusted for sex, age, and height using the Global Lung Function Initiative (GLI)-2012 equations DSST score, Digit symbol substitution test; TMT-A and -B, Trail Making Tests, part A and B; PSS, Perceived stress score; WHOQOL-Bref, World Health Organization Quality of Life – BREF; TBARS, Thiobarbituric acid reactive substances; SOD, Superoxide dismutase; MDA, Malondialdehyde; GSH, Glutathione. The adjusted means of all variables are presented along with standard errors (SE)*.

*Adjusted means stand for the mean values of the outcome variables adjusted for covariates age, educational level, BMI, cumulative exposure index (CEI), and serum achetylcholinesatse levels for raw spirometric variables. However, for Global Lung Function Initiative (GLI) percent predicted variables, age and BMI adjustments were not done as the data is standardized for age, height and ethnicity. AMD: Adjusted mean difference, differences in the adjusted means between the two groups (i.e., adjusted for the covariate)*.

* *Indicate P-value < 0.05, and*

** *indicate P < 0.01*.

*GLI z-scores are independent of age, and height*.

In exploratory subgroup analyses, greater improvements in spirometric variables were noted in farmers with age>39 years as compared to those ≤39 years (data not shown).

### Secondary Outcomes

The secondary variables were the cognitive and psychological variables. The post-intervention mean scores of DSST [AMD = 11.82 (95% CI, 8.90–14.75)], TMT-A [AMD = −24.60 (95% CI, −28.14 to −21.05)] and TMT-B [−41.99 (−49.72 to −34.25)] were significantly improved in the yoga group as compared to the control group (Table 2). The influence of BFY on the contrive outcomes was not confounded by age or education (Table 2). We could also observe significant improvement in WHO-BREF scores as compared to the control group [AMD = 26.89, (95% CI = 22.82–30.97)]. Concerning PSS, positive but non-significant changes in the adjusted means were observed between BFY and the control group (Table 2).

### Mediation Analysis

#### Test of Direct Effect of Treatment on the Mediators

Concerning the proposed mediators of yoga intervention, MDA demonstrated a significant reduction in the yoga group as compared to the control group [(AMD = −63.72, 95% CI = −91.94– (−35.05)], whereas the anti-oxidative markers GSH and SOD indicated a comparative increase in the yoga group [AMD; GSH = 1.08, 95% CI = 0.79–1.37; AMD; SOD = 0.06, 95% CI = 0.010–0.11] as compared to the controls (Table 2). Hence, significant associations could be established between BFY and all the potential mediators using linear regression models (path a, Supplementary Figure 1). Therefore, further mediation models as presented in Table 3 were fitted to all the three (SOD, MDA, and GSH) variables.

**Table 3:** Indirect, direct, and total effects of the mediation models on respiratory and cognitive outcomes at 6 months.

**Outcomes**	**Mediators**	**Indirect effects (mediating-effect)**	**Direct effect of BFY intervention on outcome (DE)**	**Total effect (TE)**	**Proportion mediated (%)**
FEV(L)	SOD	0.02 (−0.03–0.09)	1.06 (0.67–1.45)**	1.01 (0.74–1.29)**	1.98
	MDA	−0.08 (0.07–0.24)			7.92
	GSH	0.01 (0.11–0.21)			1.00
FEV z score	SOD	0.018 (−0.11–0.15)	1.90 (1.11–2.69)	1.78 (1.24–2.33)**	1.01
	MDA	−0.15 (−0.50–0.15)			8.43
	GSH	0.01 (−0.45–0.48)			0.56
GLFEVPP	SOD	0.02 (−0.03–0.09)	47.68 (33.93–61.43)**	48.64 (38.84–58.44)**	0.04
	MDA	1.08 (−3.53–5.83)			2.22
	GSH	1.11 (−6.74–8.03)			2.28
FVC(L)	SOD	0.024 (−0.044–0.11)	1.30 (0.81–1.80)*	1.12 (0.76–1.48)*	2.14
	MDA	−0.16 (−0.36–0.018)			14.28
	GSH	−0.045 (−0.32–0.21)			4.02
FVC z score	SOD	0.01 (−0.06–1.00)	2.32 (1.34–3.29)	2.03 (1.34–2.71)	2.14
	MDA	−0.14 (−0.34–0.02)			14.28
	GSH	−0.045 (−0.32–0.21)			4.02
GLFVCPP	SOD	−1.29 (−3.99–0.48)	49.58 (35.60–63.59)*	46.47 (36.49–56.45)*	3.00
	MDA	−0.92 (−5.83–3.70)			2.00
	GSH	0.89 (−8.07–5.46)			1.91
FEV1/FVC	SOD	0.27 (−0.42–1.22)	−1.36 (−6.80–4.08)	2.54 (−1.43–6.50)	10.63
	MDA	2.31 (0.68–4.51)			92.03
	GSH	1.31 (−1.18–3.82)			0.52
GLIFEV1/FVCPP	SOD	0.04 (−0.79–0.95)	−3.15 (−8.54–2.24)	0.49 (−3.35–4.34)	8.16
	MDA	2.96 (0.85–5.38)			–
	GSH	0.64 (−2.37–3.48)			–
FEV1/FVC z-Score	GSH	0.00 (−0.11–0.13)	−0.55 (−1.24–0.14)	−0.04 (−0.54–0.45)	–
	MDA	0.40 (0.15–0.13)			–
	GSH	0.10 (−0.30–0.46)			–
FEF25–75%	SOD	0.28 (−1.07–2.02)	18.78 (9.40–28.17)*	28.39 (21.49–35.29)*	0.99
	MDA	2.51 (−0.23–5.75)			8.84
	GSH	6.80 (1.51–12.36)			23.95*
FEF25–75% z-Score	SOD	0.03 (−0.33–0.45)	4.72 (2.36–7.07)	6.94 (5.34–8.5)*	0.004
	MDA	0.83 (−0.07–1.80)			11.95
	GSH	1.60 (0.48–2.90)			23.50*
PEFR	SOD	1.43(−0.43–4.02)	43.64 (31.90–55.38)*	44.47 (36.15–52.79)*	3.21
	MDA	1.60 (−1.22–4.95)			3.59
	GSH	−0.72 (−6.41–4.90)			1.62
DSST	SOD	0.11 (−0.69–0.93)	10.83 (6.74–14.92)*	11.71 (8.70–14.73)*	0.94
	MDA	0.026 (−1.39–1.51)			0.26
	GSH	0.71 (−1.23–2.70)			6.06
TMT-A	SOD	0.24 (−0.76–1.54)	−24.25 [−28.91 –(19.60)]*	−24.60 [−28.13– (−21.07)] *	0.99
	MDA	0.40 (−1.16–1.78)			8.69
	GSH	−0.96 (−3.60–1.41)			3.90
TMT-B	SOD	1.58(−0.39–4.07)	−44.36 (−54.81– −33.91)*	−42.40 (−50.28– −34.51)*	3.73
	MDA	1.28 (−1.81–4.75)			3.02
	GSH	−0.82 (−6.31–4.87)			1.93
PSS	SOD	−0.06 (−0.63–0.45)	−3.60 (−6.19– −1.01)	−1.63 (−3.51–0.25)	3.92
	MDA	0.52 (−0.40–1.51)			31.91
	GSH	1.46 (0.15–3.02)			89.57
WHOQOL-Bref	SOD	−0.61 (−1.98–0.25)	26.07 (20.72–31.41)	27.61 (23.61–.61.63)*	2.21
	MDA	−0.26 (−1.71–1.12)			0.94
	GSH	2.41 (−0.57–5.86)			8.72

*Direct effect (DE) refers to the direct influence of the BFY on the study outcomes that is not mediated by other variables in the model. An indirect (mediated) effect expresses the portion of the intervention effect that is mediated through a specific mediator. The total effect (TE) is the sum of the direct and indirect effects of the BFY and of mediators on the study outcomes*.

*SOD, Superoxide dismutase; MDA, Malondialdehyde; GSH, Glutathione; FEV, Forced expiraory volume; FVC, Forced vital capacity; PEFR. GLGlobal Lung Initiative (GLI) percent predicted (pp), and GLI z-scores, The adjusted means of all variables are presented along with standard errors. Adjusted for age, BMI, and education (yrs), adjusted for respiratory symptoms*.

#### Test of the Indirect (Mediating) Effect

The indirect, direct and total effects of each of the models are given in Table 3. The mediation analyses indicated GSH as a mediator of the effect of BFY on FEF5-5. As observed in Table 3, a fraction of FEF25-75 change was partly explained by increases in GSH levels (mediation percentage 23.95%).

## Discussion

In this 24-weeks randomized controlled trial on chronically pesticide exposed farmers, BFY practice was significantly more observed to be more effective than the wait-list control condition in the alleviation of spirometry-based indices of airflow limitation, in particular FEV1, FVC, FEV25-75, and PEFR. The observed increment in FEV1 by 1.02L over 6 months in the BFY group seems relevant against an annual decline by 13.1 mL (95% CI, 19.1 to 7.1) (7) and a reduction by 140 ml observed over an average of 3.4 years of pesticide exposure (10). However, given the lack of specific reports on clinical interventions with spirometry-based pulmonary outcomes in pesticide-exposed populations, there remains an uncertainty in the clinical significance of the observed effect sizes. Nonetheless, the observed change of ~1 l in FEV1 is larger than the minimal clinically important difference of 100 ml suggested for pharmacological trials (56). Our observations accord with the previous reports of improvements in pulmonary function parameters with regular yoga practice, particularly breathing-focused practices (18–20). Additionally, there have been mixed findings as well-indicating that the effectiveness of yoga-based breathing interventions is influenced by the fitness levels of the subjects, with only marginal improvements in lung functions observed in the elderly (20) to moderate-but-clinically-significant improvements in COPD patients (18). This further explains the comparatively larger effect-sizes observed concerning FEV1 and FEV1 (Pred%) in the present pesticide-exposed cohort as compared to the meta-analyzed effect-sizes on patients with COPD [weighted mean difference (WMD) of 125 ml for FEV1(L)20 and 3.95% for FEV (Pred%)] (18). Pesticide exposure has been sought as a risk factor for obstructive pulmonary diseases marked by an early reduction in FEV1 (57). Our results justify the relevance of early intervention in pesticide-exposed populations for prevention of manifestations of irreversible lung function decline as in COPD (57). Mechanistically, we could establish a 24% mediating effect of glutathione increment underlying BFY induced improvements in FEF25-75, which is another primary spirometry-based marker of the airway in abundance in obstruction (4). Glutathione is the principal small molecular weight thiol of the antioxidant system abundant in the epithelial lining fluid of lungs and serves as a crucial protector of alveolar macrophages, pulmonary epithelial cells, and pulmonary endothelial cells from oxidative stresses (33). Its depletion and disturbed metabolism are key manifestations in pesticide exposed tissues under inflammatory settings of lung decline including chronic obstructive pulmonary disease (COPD). Our findings on GSH augmentation accord with prior reports on remarkably increased after yoga practice serum total glutathione (GSH) contents, activities of GSH-peroxidase, and GSH-transferase (58). The notion BFY could serve as a non-pharmacological substitute for GSH augmentation deserves attention since supplementation of GSH precursors has been considered as the best means of manipulating intracellular GSH biosynthesis to combat its depletion noted in varied inflammatory settings (59). For other spirometric parameters we failed to establish a significant mediating effect of alleviation of oxidative stress on BBY intervention. These findings indicate the need to explore other alternate markers, including inflammation. Altogether, the observed beneficial effects of BFY on FEV1 along with other spirometric markers of small airway obstruction (FEV25-75% and PEFR) deserve clinical attention to combat exacerbations of lung function decline in pesticide-exposed populations. These findings deserve clinical recognition given the observed poor status of precautionary practices in the farming population; most of the farmers (*n* = 102, 72.85%) were not using personal protective equipment. Moreover, when analyzed for airflow obstruction, 56.3% had airflow obstruction and ~70% of the farmers reported adverse respiratory symptoms.

Pesticides are known lipophilic neurotoxins and are reported to cross and disrupt the blood-brain barrier (60). Long-term exposure to these chemicals could lead to neuronal loss in specific brain regions and subsequent cognitive impairment (61). In line with previous reports on pesticide exposure and global cognitive function, the entire cohort of pesticide-exposed farmers had the manifestation of mild cognitive impairment (MOCA scores 18–25) (14, 33). In particular, the TMT-B scores of the study cohort were lower as compared to the normative population-based scores reported for the matched age and education status, indicative of their compromised executive control (61, 62). In this backdrop of cognitive decline, BBY intervention was found to have significant potential to mitigate neurocognitive decline through improvements in the TMT-B scores by 42 s, DSST by 11 s, and TMT-A by 25 s. Our results are in line with previous reports of yoga-based practices, However, no causal inference could be established for oxidative stress markers underlying the beneficial cognitive effects of the BFY. Inclusion of the objective mediators such as structural and functional brain changes could aid in unraveling the mediator influences.

Psychological stress is a well-recognized health concern amongst farmers. Though we could find a trend for improvement in perceived stress in the BFY group, the difference between the groups was not statistically significant. Notably, there was a significant improvement in the quality of life in the BFY group as compared to the control group, which is a positive health marker indicative of improved capacity to function (63). and an important factor toward the attainment of sustainable agriculture (64).

This study is limited by the small sample size and use of prebronchodilator spirometry. The study was focused on early intervention in the high-risk farming population, the trial was of a short duration of 6 months, and hence, we did not include the outcome of COPD manifestation which would be required to get a more realistic insight into the preventive potential of BFY. We did not consider statistical power requirements for causal analyses which need extended validation in larger trials (29). The strength of this study lies in the fact that it is the first-ever trial that addressed the need for clinical attention to alleviate adverse health conditions in the chronically pesticide-exposed farmer population. The present trial was conducted in India which is predominantly a rural country with 67% of its population engaged in agricultural practice (65). In the Indian scenario, farmers mainly live in rural areas wherein government hospitals are the major health care setups with a preponderance of traditional health experts (66). However, originating in India as a comprehensive mind-body practice, yoga has become increasingly popular in the West as a holistic approach to health and well-being, the popularity and practice of yoga-based interventions are not restricted to the Indian subcontinent (67, 68). Over recent years, there has been a sharp rise in the spread of yoga-based interventions across the globe. Given the fact that ethnicity is an important factor in lung function, the trial findings need validation over different ethnic settings. Overall the study findings are useful for establishing preliminary evidence for future larger trials with longer follow-ups targeting the prevention of COPD in the high-risk population.

Enhanced respiratory surveillance has been stated as a need of the hour for pesticide-exposed farmers. Our findings indicate the scope of implementation of cost-effective breathing-focused interventions along with respiratory surveillance in pesticide exposed farmers. Given the multimodal influence of yoga on health, the effects of yoga may be broader when explored for other adverse health effects associated with pesticide exposure. Overall, the findings support the use of yoga-based interventions as a pragmatic strategy against exacerbations of respiratory and cognitive health decline in farming communities. In this 24-weeks randomized controlled trial on chronically pesticide exposed farmers, breathing focused yoga intervention was significantly more effective than the wait-list control condition in the alleviation of spirometry-based indices of airflow limitation, in particular FEV1, FEV25-75, and PEFR. The study also gave mechanistic insights into the understanding of the breathing-focused yoga intervention vis GSH augmentation for improvement for FEF5-5%. This could serve as a cost-effective substitute for GSH supplementation suggested for the management of airway inflammation.

## Data Availability Statement

The raw data supporting the conclusions of this article will be made available by the authors, without undue reservation.

## Ethics Statement

The study was conducted in accordance with the CONSORT statement for non-pharmacological interventions and was approved by the Institutional Ethics Committee. The patients/participants provided their written informed consent to participate in this study.

## Author Contributions

VD: conceptualization, study design, and data analysis. VM: conceptualization, study design, data analysis, writing first draft of manuscript, and revision of manuscript. NM: conceptualization, study design, and revision of manuscript. US: data analysis. DS: revision of manuscript. All authors contributed to the article and approved the submitted version.

## Conflict of Interest

The authors declare that the research was conducted in the absence of any commercial or financial relationships that could be construed as a potential conflict of interest.

## Publisher's Note

All claims expressed in this article are solely those of the authors and do not necessarily represent those of their affiliated organizations, or those of the publisher, the editors and the reviewers. Any product that may be evaluated in this article, or claim that may be made by its manufacturer, is not guaranteed or endorsed by the publisher.
