# Supplementary material for: Breathing-Focused Yoga Intervention on Respiratory Decline in Chronically Pesticide-Exposed Farmers: A Randomized Controlled Trial
Source: Front Med (Lausanne). 2022 Mar 11;9:807612. doi: 10.3389/fmed.2022.807612 (PMC8965718; doi:10.3389/fmed.2022.807612)
Supplement: Supplementary file 1 [file Data_sheet_1.docx]

STUDY PROTOCOL: EFFECT OF BREATHING FOCUSED YOGA INTERVENTION ON ADVERSE RESPIRATORY AND COGNITIVE AMONG AGRICULTURAL FARMERS OCCUPATIONALLY EXPOSED TO PESTICIDES

**INTRODUCTION**

In India, government hospitals are the primary source of health care in rural settings, where medical staffs are usually traditional Indian medicine (Ayurvedic, homeopathic, and Ayush) practitioners.^1,2^ Yoga is one of the widely popular Ayush-based interventions in India and across the world with high general acceptability;^3,4^ approximately 11.8% of the Indian population have been reported to be practicing yoga across both rural and urban locations of the country.^3^ As a multi-component health intervention Yoga has been associated with wide-ranging health effects under physical and mental health domain under varied clinical settings.^5^ A meta-analyses reported beneficial impact of yoga training on improvement of FEV_1_ in patients with chronic obstructive pulmonary disease (COPD) [weighted mean difference (WMD): 123.57 mL, 95% CI: 4.12-243, P=0.04], FEV_1_% pred (WMD: 3.90%, 95% CI: 2.27-5.54, P<0.00001), and 6 MWD (WMD: 38.84 m, 95% CI: 15.52-62.16, P=0.001).^6^ The available evidence from RCTs on Yoga also indicates improvements in cognitive function with moderate effect size.^7,8^ A meta-analysis of both short- and long- term effects indicates that yoga practice is associated with improvement in cognitive functioning generally in both long-term (Hedges’ g = 0.33) and short-term studies (Hedges’ g = 0.56), with medium effect sizes reported in short-term studies’ measures of attention and processing speed (Hedges’ g = 0.49) and executive functioning (Hedges’ g = 0.39)^68^ These lines of evidence provided the rationale for testing the efficacy of yoga-based interventions in long-term pesticide exposed farmers against the adverse health effects of pesticide exposure, in particular, lung and cognitive functions. Hence, we aim to conduct a two armed randomized clinical trial of 6 months to test the hypothesis that yoga-based intervention is effective in preventing the worsening of lung and cognitive function in pesticide-exposed farmers against a wait-list control group. Identifying of the underlying mechanisms that could lead to an intervention related improvement in outcome is one of the major necessities to eatbslish the efficacy of interventions.^9^ Here we hypothesize that oxidative stress could be the mediating mechanisms underlying the plausible.

**Sample size**

Based on the assumption of an effect size of 0.54 for FEV1 as derived from a prior report, a sample size of 140 subjects was derived to obtain a power of 80% and a 2-sided α =0.05, with an assumed attrition of 20% over 6 months.

**Randomization and blinding**

An external statistician, not directly involved in implementation of the BFY had randomized the participants during their baseline visit in a 1:1 ratio (n=70, each arm) using a sequence randomizer. The allocation sequences were sealed and participants were informed about the further process immediately after their baseline assessments. Owing to the nature of the intervention, blinding was not possible, however, outcome measures were blinded for the randomisation groups.

**Study design and participants**

The study will be a two armed, randomized, parallel group trial with yoga-based intervention and wait-list control groups. To our knowledge, this is the first study examining the efficacy of BFY on the management of adverse health problems in pesticide-exposed farmers. The study was conducted at district Panipat, State Haryana located in the Northern part of India. Five agriculture prominent villages were covered namely Ujah, Ugra Kheri, Jalpar, Risalu, and Dhan soli. Farmers were invited to participate and were recruited during the meetings organised by the village organisation called panchayat A rough sampling frame of 500 households was generated (100 per village). Farmers were invited to participate and were recruited during the meetings organised by the village organisation called panchayat. Door-to-door visits were made afterwards to screen the households for farmers who self-reported pesticide use of at least 6 months. From each household only one participant was selected and details were obtained on the crops that they usually work on and their exposure to pesticides After a detailed explanation of the study objectives and design, informed consent was obtained from willing individuals. The study was conducted in accordance with the CONSORT statement for non-pharmacological interventions and was approved by the Institutional ethics committee of SVYASA, Bengaluru, India RES/IEC-SVYASA/148/2019*.* The study will be registered with clinical trial registry of India. The study participants will be male farmers of age group between 18-49 years, with at least at least 6 months of self-reported spraying operations in the field. Details of inclusion and exclusion are listed below in Supplementary Table 1.

**Supplementary Table 1.**  Detailed inclusion and exclusion criteria

| **Inclusion criteria** |
| --- |
| Male gender |
| Aged 18-49 years |
| Chronic pesticide exposure < 6 months of spraying duration |
| **Exclusion criteria** |
| Chronic rhinitis, with partial or complete obstruction of one or both nostrils |
| Current or ex-smokers with a smoking history of ≥10 pack-years |
| Established history of COPD, current diagnosis of asthma or respiratory disorders |
| Any life-threatening condition with life expectancy <3 years, other than vascular disease or COPD, that might prevent the subject from completing the study |
| Prior exposure to yoga or any other mind-body medicine |
| Previous knowledge of and training in yoga respiratory exercises, inability to comply with the protocol (not attending >40% of classes), presence of cardiovascular or any other diseases, and use of medication that could affect autonomic modulation of the heart. |
| Symptoms of acute pesticide exposure/poisoning |

**Supplementary Table 2. Details of intervention adapted from protocol developed by Santaella et ., 2011.^10^**

| Aim | The intervention isadopted from the protocol reported by Santella et al. It was aimed for improving lung and cognitive functions |
| --- | --- |
| Yoga-trainers | Certified yoga therapists delivered the sessions. |
| Format | Initial 3 months of intervenion was given 45 minutes per day for 6 days in a week, spread over a period of 24 weeks, interrupted by weekend breaks of one day and will be advised to practice the same on their own on the off days. Follwing the same, home-based practice was advised for next 3 monts. The course was given to a group of 15-20 patients at a time |
| Key elements of intervention | Intervention would be an integrated yoga based lifestyle protocol customised for geriatric population adapted from our previous study. Same protocol was maintained throughout the study to avoid the risk of intervention variability. Intervention also included weekly once group lectures focused on importance of wearing personal protective equipments during pesticide spray |
| Detailed content of intervention | The yoga intervention will be based on Hatha Yoga that included physical postures (asanas), breathing exercises and moderate meditation. Subjects will practice 2-min savasana (relaxation) followed by preparatory physical postures and voluntarily regulated fast breathing (Bhastrika pranayama, 25 min) and yoga-based guided relaxation (15 min)  Savasana- 2min  Preparatory physical postures- 5 min   - Pavanamuktasana (Wind-Relieving Pose) - Sukhasana (Easy Pose) - Gomukhasana (Cow Face Pose) - paschimotanasana(back-stretching posture) - vakrasana(sitting sideward twist posture)   Pranayama-25 min  The practice of the bhastrika pranayama was adapted from ; each round of the practice is composed by a set of fast breathing (kapalabhati) followed by a slow inspiration through the right nostril, a comfortable apnea done with the three bandhas (mula, jalandhara, and uddiyana) and a slow expiration through the left nostril (Surya bedhana). The relation inspiration:apnea:expiration was set according to individual comfort, varying from 1:1:2 to 1:2:2; 1:3:2, or 1:4:2—apnea never exceeded four times the inspiration time, and expiration was set to constantly correspond to twice the inspiration time.  Relaxation-10-15 min |
|  |  |

**Ethical consideration**

The project shall be reviewed by the institutional ethical committee (IEC) and informed consent will be obtained from each participant after informing trail of the research to everyone.

***Outcomes and measures***

The primary outcome of this trial are the spirometry based respiratory variables. Assessments will be made before the trial and after 6 months post-intervention. Our outcomes are proposed to test whether the intervention has the potential to improve the status of lung function assessed through spirometry-based variables. Secondary variables would include markers of cognitive function, digit symbol substitution test and trial making tests part A and B.

**Statistical Methods**

All randomised patients will be included in the analysis in accordance with the intention-to-treat analysis principle. Comparisons between the groups on continuous primary and secondary endpoints will be performed using a linear model including intervention groups as a factor and the baseline values as the covariates (where applicable).

**Pesticide exposure Index**

The algorithm for cumulative pesticide exposure index was used as described by Lee et al. Using the formula, CEI = cumulative index= Intensity level × spraying year × spraying day per year. Following algorithm were used to scoring PPE^12,13^

**Supplementary Table 3: Algorithm score factors for personal protective equipment (PPE)**

| **PPE3** | **PPE2** | **PPE1** | **PPE0** | **Overall PPE Score** |
| --- | --- | --- | --- | --- |
| Yes | Yes | Yes | No | 0.1 |
| Yes | Yes | No | No | 0.3 |
| Yes | No | Yes | No | 0.4 |
| No | Yes | Yes | No | 0.5 |
| Yes | No | No | No | 0.6 |
| No | Yes | No | No | 0.7 |
| No | No | Yes | No | 0.8 |
| No | No | No | Yes | 1.0 |

PPE-3: Chemically resistant rubber gloves, PPE-2: Cartridge respirator or gas mask, Disposable outer clothing, PPE-1: Face shields or goggles, Fabric/leather gloves, Other protective clothing, such as boot, PPE-0: PPE not used

**Supplementary Table 4**. Characteristic distributions between participants and non-participants

| **Variable** | **Participants**  **(n=144)** | **Non-participants**  **(n=136)** | **Test statistic** | **P value** |
| --- | --- | --- | --- | --- |
|  |  |  |  |  |
| Age, years | 38.54 (7.49) | 35.96 (10.84) | 2.33 | <0.001 |
| Pesticide exposure, years | 5.50 (3.30) | 3.64 (1.90) | 5.69 | <0.001 |
| Adverse respiratory symptoms |  |  |  |  |
| Wheezing | 22 (15.7) | 19 (1.97) | 3.19 | 0.366 |
| Dry Cough | 12(8.6) | 14 (8.90) |  |  |
| Productive Cough | 106 (75.7) | 65 (47.77) |  |  |
| Dyspnoea | 98 (70%) | 77 (56.62) |  |  |

When analyzed for distribution of demographic parameters, participants were older as compared to non participants, with significantly higher exposure to yoga as compared to controls [5.50(3.30) vs. 3.64 (1.90)] years. Similarly, there was a higher distribution of adverse respiratory symptoms in yoga group compared to controls .

**Supplementary** **Table 5. Distribution of different types of pesticides amongst the farmers**

| ORGANOPHOSPHATE (70%) | Monocrotophos,Chlorpyrifos,Profenofos,Acephate |
| --- | --- |
| NEONICOTINOID  (10%) | Confidor,Acetamipride powder |
| PHOSPHANOGLYCINE (10%) | Round up |
| PYRETHROID (10%) | Metaphor,Topic,Leader  ` |

**Supplementary Table 6. Distribution of pesticide handling and equipment use related parameters between intervention and control groups**

|  | **Variable** | **Total**  **(n=140)** | **Yoga**  **(n=70)** | **Control**  **(n=70)** | **Test** | **P value** |
| --- | --- | --- | --- | --- | --- | --- |
| 1. | **Handling of pesticides, n (%)** |  |  |  |  |  |
|  | Bare hands, | 124 (88.6) | 55 (78.6) | 69 (98.6) | 0.559 | 1.00 |
|  | Measuring cups | 14 (10.0) | 13 (18.6) | 1 (1.4) |  |  |
|  | Others | 2 (1.4) | 2 (2.9) | 0 (0) |  |  |
| 2 | **Method of spray, n (%)** |  |  |  | 9.84 | 0.024* |
|  | Handspray | 101 (72.1) | 53 (75.7) | 48 (68.6) |  |  |
|  | Handspray, backpack | 31(22.1) | 10 (14.3) | 21 (30.0) |  |  |
|  | Handspray, motor spray | 3(2.1) | 2(2.9) | 1(1.4) |  |  |
|  | Handspray, backpack, motorspray | 5(3.6) | 5(7.1) | 0(0) |  |  |
| 3 | **Personal protective equipment** (PPE) |  |  |  | 117.962 | <0.001 |
|  | Boots, cap | 10 (7.14) | 1 (1.42) | 9 (12.85) |  |  |
|  | Cap, mask | 5 (0.71) | 4 (0) | 1 (1.42) |  |  |
|  | Cap, mask, gloves | 1 (0.71) | 1 (1.42) | 0 (0) |  |  |
|  | Gloves, cap | 1 (0.71) | 0 (0) | 1 (1.42) |  |  |
|  | Mask | 21 (15.00) | 5 (7.14) | 16 (22.85) |  |  |
|  | None | 102 (72.85) | 59 (84.28) | 43 (61.42) |  |  |
| 4 | **Food ingestion during spray** | 25 (17.85) | 7 (10.00) | 18 (25.71) | 5.892^a^ | 0.013* |
| 5 | **Washing hands post spray** | 20 (14.2) | 8 (11.42) | 12 (17.14) | 0.933^a^ | 0.235 |
| 6 | **Washing of hands before eating** food | 16 (11.42) | 4 (5.71) | 12 (17.14) | 4.516^a^ | 0.030 |

Continuous variables are represented as means (SD), and categorical variables are represented as number (%); t= independent samples t-test statistic, and χ^2^= Chi-Square test statistic

During pesticide spraying season these pesticide sprayers were exposed to a mixture of various types of pesticides. Based on type of crop pesticide is sprayed. Types of pesticides exposure depend upon the class of organisms designed to control (weedicide, fungicide, rodenticide, and herbicide) or by the chemical structure (organochlorine, organophosphates, carbamates, chlorinated hydrocarbons, and dipyridyls). Round up (Isopropylamine salt of N-(phosphonomethyl) glycine), profenfos (O-4-bromo-2-chlorophenyl-O-ethyl S-propyl phosphorothioate), and dichlorovos (2,2-Dichlorovinyl dimethyl phosphate) are widely used organophosphates in India. The most commonaly used pesticides were monocrotophos, chlorpyrifos, profenofos, acephate, confidor, acetamiprid, round up, matador, topic and leader.

Pulmonary function measures were also evaluated for association with obstructive or restrictive lung disease. 48 farmers (34.3%) had FVC measures less than 80% of predicted, 8 (7.0%) had FEV1 less than 80% of predicted, and 9 (7.9%) had a FEV1/FVC ratio.

-63.804 (-92.15--35.45)

**Supplementray Table 7.** The key concepts and target causal effects used in the mediation analysis

|  |  | |  | **Adjusted mean differences** | | | | | |  | | |  | | |  | |  |  |
| --- | --- | --- | --- | --- | --- | --- | --- | --- | --- | --- | --- | --- | --- | --- | --- | --- | --- | --- | --- |
| **Target effect** | **Definition****[*](https://www.sciencedirect.com/science/article/pii/S0895435621000512?dgcid=author#tb1fn1)** | |  | **MDA** | | | | | | **GSH** | | | **SOD** | | |  | |  |  |
| Total effect | The average difference in the outcome between individuals allocated to the yoga intervention versus control group | |  |  | | | | | |  | | |  | | |  | |  |  |
| ^a^Intervention–mediator effect | The average difference in the mediator between individuals allocated to the intervention versus control groups | |  | -63.80 (-92.15--35.45)* | | | | | | 56.14, 0.32* | 4.89, 0.04 |  | | |  |  |  |  |  |
|  |  | |  |  | | | | | |  |  | | | | |  | |  |  |
| ^b^Mediator–outcome effect | |  | | | | |  |  | |  | | | |  | | | | |  |
| FVC | | | | |  | 0.97, 0.02 | | | 17.20, 0.23* | | 1.52, 0.03 | | | | |  |  |  |  |
| FVCL | | | | |  | 0.45, 0.01 | | | 6.92, 0.11* | | 2.15, 0.04 | | | | |  |  |  |  |
| FEV1L | | | | |  | 0.20, 0.00 | | | 0.16, 0.00 | | 2.18, 0.04 | | | | |  |  |  |  |
| FEV1 | | | | |  | 2.25, 0.04 | | | 18.83, 0.25* | | 1.33, 0.02 | | | | |  |  |  |  |
| FEV1/FVC% | | | | |  | 6.67, 0.10* | | | 0.18, 0.00 | | 0.41, 0.01 | | | | |  |  |  |  |
| FEV25-75 | | | | |  | 2.62, 0.04 | | | 20.14, 0.26** | | 1.44, 0.02 | | | | |  |  |  |  |
| PEFR | | | | |  | 1.33, 0.01 | | | 15.33, 0.21** | | 1.89, 0.03 | | | | |  |  |  |  |
| DSST | | | | |  | 3.77, 0.06* | | | 7.45, 0.012* | | 2.24, 0.04 | | | | |  |  |  |  |
|  | TMT-A | | | |  |  | | | 23.75, 0.29** | | 0.39, 0.01 | | | | |  |  |  |  |
|  | TMT-B | | | |  | 1.06, 0.02 | | | 13.62, 0.19** | | 0.04, 0.001 | | | | |  |  |  |  |
| Indirect effect | The average difference in the outcome caused by the average effect of the intervention on the mediator | | | |  | Mentioned in table 4. | | | Mentioned in table 4. | | Mentioned in table 4. | | | | |  |  |  |  |
| Direct effect | The average difference in the outcome caused by the intervention while the mediator is held to its natural level under the control or the intervention | | | |  | Mentioned in table 4. | | | Mentioned in table 4. | | Mentioned in table 4. | | | | |  |  |  |  |
|  |  | | | |  |  | | |  | |  | | | | |  |  |  |  |

All estimates are presented with F values and Eta square values; a explains the relationships between treatment allocation and change in each of the potential mediators, b represents the average difference in the outcome across different levels of the mediator

**Supplementary Fig 1.** Mediation model


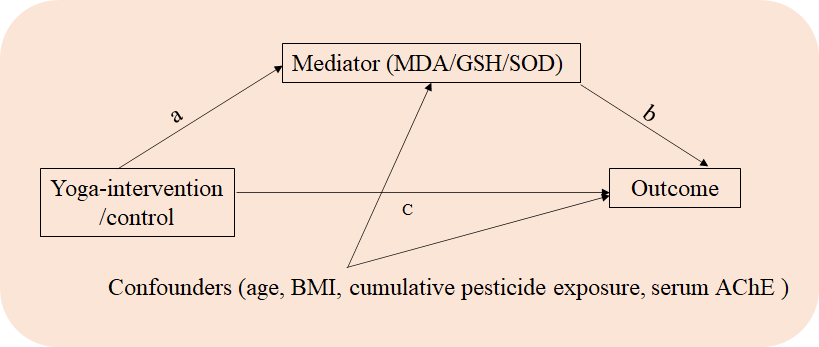


Outcomes were reported for each model including point estimates, confidence interval and significance levels of path a, path b, total effect, direct effect, indirect effect, and proportion mediated. An indirect effect was reported as significant if the 95% confidence interval did not include 0. Linear regression analyses were performed to examine the relationships between treatment allocation and change in each of the potential mediators, and between change in each of the potential mediators and the outcome posttreatment score. This provided tests of the a and b paths separately

Additional References

1. Abhilash PC, Singh N. Pesticide use and application: an Indian scenario. J Hazard Mater. 2009 Jun 15;165:1-12. doi: 10.1016/j.jhazmat.2008.10.061.
2. Bawaskar PH, Bawaskar PH, Bawakar HS. India--small progress in health care, decline in rural service. Lancet. 2015;386:2389. doi: 10.1016/S0140-6736(15)01189-7.
3. Mishra AS, Sk R, Hs V, Nagarathna R, Anand A, Bhutani H, Knowledge, Attitude, and Practice of Yoga in Rural and Urban India, KAPY 2017: A Nationwide Cluster Sample Survey. Medicines (Basel). 2020;7:8. doi: 10.3390/medicines7020008. PMID: 32033426; PMC7168227.
4. Chattopadhyay K, Mishra P, Manjunath NK, Harris T, Hamer M, Greenfield SM,. Development of a Yoga Program for Type-2 Diabetes Prevention (YOGA-DP) Among High-Risk People in India. Front Public Health. 2020;8:548674.
5. Büssing A, Michalsen A, Khalsa SB, Telles S, Sherman KJ. Effects of yoga on mental and physical health: a short summary of reviews. Evid Based Complement Alternat Med. 2012;2012:165410.
6. Liu XC, Pan L, Hu Q, Dong WP, Yan JH, Dong L. Effects of yoga training in patients with chronic obstructive pulmonary disease: a systematic review and meta-analysis. J Thorac Dis. 2014 ;6:795-802. doi: 10.3978/j.issn.2072-1439.2014.06.05.
7. Gothe NP, McAuley E. Yoga and Cognition: A Meta-Analysis of Chronic and Acute Effects. Psychosomatic medicine. 2015;77:784–97.
8. Brenes GA, Sohl S, Wells RE, Befus D, Campos CL, Danhauer SC. The Effects of Yoga on Patients with Mild Cognitive Impairment and Dementia: A Scoping Review. Am J Geriatr Psychiatry. 2019;27:188-197. doi: 10.1016/j.jagp.2018.10.013.
9. Bernardi L, Porta C, Gabutti A, Spicuzza L, Sleight P. Modulatory effects of respiration. Auton Neurosci. 2001 Jul 20;90(1-2):47-56. doi: 10.1016/S1566-0702(01)00267-3. PMID: 11485292.
10. Santaella DF, Devesa CR, Rojo MR, Amato MB, Drager LF, Casali KR, et al. Yoga respiratory training improves respiratory function and cardiac sympathovagal balance in elderly subjects: a randomised controlled trial. BMJ Open. 2011 May 24;1:e000085.
11. Ellman GL. Tissue sulfhydryl groups. Archives of biochemistry and biophysics 1959; 82: 70-7.
12. Chambers JE, Chambers HW. An investigation of acetylcholinesterase ibhibition and aging and choline acetyltransferease activity following a high level acute exposure to paraoxon. *Pest Biochem Physiol.*1989;33:125–31.
13. American Thoracic Society – Standardization of spirometry 1995 update. *Am J respire Crit Care Med.*1995;152:1107–36.
14. *Wechsler, D. Adult Intelligence Scale- revised*. New York: Psychological Corporation, 1981.
15. Matarazzo JD, Herman D. Base rate data for the WAIS-R: test-retest stability and VIQ-PIQ differences. *J Clin Neuropsychol* 1984; 6: 351–66.
16. Jaeger J. Digit Symbol Substitution Test: The Case for Sensitivity Over Specificity in Neuropsychological Testing. J Clin Psychopharmacol. 2018t;38:513-519.
17. Bowie C.R., Harvey P.D. Administration and interpretation of the Trail Making Test. *Nat. Protoc.*2006;**1**:2277–2281. doi: 10.1038/nprot.2006.390.
18. Reitan R. Validity of the Trail Making Test as an indicator of organic brain damage. Percept Mot Skills. 1958; **8** : 271 -6
19. Olivera-Souza RD, Moll J, Passman LJ, Cunha FC, Paes F, Adriano MV, et al. Trail making and cognitive set-shifting. Arq Neuropsiquiatr. 2000; **58**(3B) : 826 -9 [[PubMed](https://www.ncbi.nlm.nih.gov/pubmed/11018818)]
20. Strauss E, Sherman EMS, Spreen O. A Compendium of Neuropsychological Tests: Administration, Norms, and Commentary. 2006;
21. Cohen S, Kamarck T, Mermelstein R. A global measure of perceived stress. J Health Soc Behav. 1983;24:385–96. pmid:666841
22. Wiriyakijja P., Porter S., Fedele S., Hodgson T., McMillan R., Shephard M., Ni Riordain R. Validation of the HADS and PSS-10 and a cross-sectional study of psychological status in patients with recurrent aphthous stomatitis. J. Oral Pathol. Med. 2020;**49**:260–270. doi: 10.1111/jop.12991.
23. Skevington SM, Lotfy M, O'Connell KA. WHOQOL Group. The World Health Organization's WHOQOL-BREF quality of life assessment: Psychometric properties and results of the international field trial. A report from the WHOQOL group. Qual Life Res. 2004;13:299–310
24. Ohkawa H, Ohishi N, Yagi K. Assay for lipid peroxides in animal tissues by thiobarbituric acid reaction. Analytical biochemistry 1979; 95: 351-8.
25. Winterbourn CC, Hawkins RE, Brian M, Carrell R. The estimation of red cell superoxide dismutase activity. The Journal of laboratory and clinical medicine 1975; 85: 337-41.
26. Thomas KW, Dosemeci M, Coble JB, Hoppin JA, Sheldon LS, Chapa G, et al. Assessment of a pesticide exposure intensity algorithm in the agricultural health study. J Expo Sci Environ Epidemiol. 2010;20:559-69. doi: 10.1038/jes.2009.54.
27. Dosemeci M, Alavanja MC, Rowland AS, Mage D, Zahm SH, Rothman N. A quantitative approach for estimating exposure to pesticides in the Agricultural Health Study. Ann Occup Hyg. 2002;46:245-60. doi: 10.1093/annhyg/mef011.
